# Somatic *GNAQ*, *CTNNB1*, and *CACNA1C* Mutations in Cat Aldosterone-Secreting Tumors

**DOI:** 10.1161/HYPERTENSIONAHA.124.23501

**Published:** 2024-10-21

**Authors:** Alice Watson, Harriet Syme, Morris Brown

**Affiliations:** Clinical Science and Services, Royal Veterinary College, London, United Kingdom (A.W., H.S.).; Clinical Pharmacology and Precision Medicine, Queen Mary University of London, United Kingdom (A.W., M.B.).

**Keywords:** aldosterone, animals, hyperaldosteronism, hypertension, mutation

## Abstract

**BACKGROUND:**

Primary aldosteronism (PA) is a common cause of human hypertension. Somatic mutations in *KCNJ5*, *CACNA1D*, *ATP1A1*, and *ATP2B3* are found in at least 80% of aldosterone-producing adenomas, which cause unilateral PA in humans. Somatic mutations have been identified infrequently in 7 other genes; few of these were known to play a role in aldosterone secretion before the discovery of their mutations. Interrogating somatic mutations in the domestic cat, in which spontaneous PA is also known to occur, might improve the understanding of normal adrenal gland physiology and the pathophysiology of PA.

**METHODS:**

DNA and RNA extracted from tissue from 13 cats with unilateral aldosterone-secreting tumors, including 8 carcinomas and 5 adenomas, underwent whole genome sequencing, targeted Sanger sequencing, and RNA sequencing. Single-nucleotide substitution variants were filtered to select those with a predicted deleterious effect on protein function and a suspected role in aldosterone secretion.

**RESULTS:**

Probable functional somatic single-nucleotide polymorphisms (n=8) were found in 3 adenomas and 2 carcinomas. Mutations with predicted significant effects were identified in 2 genes also mutated in human PA; *GNAQ* and *CTNNB1*, and in a residue of *CACNA1C* analogous to a common *CACNA1D* mutation. In contrast to humans, *CACNA1C* expression was much greater than *CACNA1D* in both feline tumor and nontumor adrenal tissue. No mutations were identified in *KCNJ5*, *CACNA1D*, *ATP1A1*, or *ATP2B3.*

**CONCLUSIONS:**

Similar mutations were identified in cats to those found in humans. It is, therefore, likely that both species have shared underlying selection pressures for mutations that increase aldosterone secretion.

**NOVELTY AND RELEVANCE**

**What Is New?**

- Somatic mutations in *CTNNB1*, *GNAQ*, and *CACNA1C* were identified in feline aldosterone-producing tumors.
- All 3 genes are the same as, or analogous to, genes with somatic mutations found in human aldosterone-producing adenomas; however, all but 1 mutation is unique to feline tumors.

**What Is Relevant?**

- Hyperaldosteronism is a cause of spontaneous hypertension in both cats and humans.
- Similar somatic mutations arose in aldosterone-producing feline tumors as in human tumors.

**Clinical/Pathophysiological Implications?**

- The spontaneous development of primary aldosteronism in a nonhuman species, with evident similarities in molecular pathogenesis, may permit prospective interventions and observations that test the role of dietary salt excess.

Primary aldosteronism (PA) is the commonest curable cause of hypertension in humans.^1,2^ It also occurs spontaneously in cats and can be cured by adrenalectomy.^3^ PA is thought to be underdiagnosed in both humans and cats.^4,5^ We have postulated that the frequency of PA in humans may be a maladaptive response to high salt intake,^6^ which is also a feature of domestic cat diets. The pathogenesis of excessive aldosterone may, therefore, be common to both species. An attraction of studying aldosterone-producing tumors in cats is their single CYP11B enzyme for aldosterone and cortisol synthesis. How a cat adrenal tumor produces only aldosterone may unmask regulators of aldosterone production that have escaped discovery in human adrenal tissue.

Germline and somatic mutations have been identified in human PA and have provided helpful clues to pathways involved in the pathogenesis of excessive aldosterone secretion.^7^ The majority of somatic mutations are in *KCNJ5* and *CACNA1D*, K^+^ and Ca^2+^ channel genes, respectively, and a further ≈10% are in *ATP1A1* and *ATP2B3*,^8^ transporters for Na^+^, K^+^, or Ca^2^^+^. Mutations of other ion-channel genes (*CACNA1H*, *CLCN2*, and SLC30A1),^9–11^ cell-adhesion molecules (*CADM1*)^12^ and G-protein subunits (*GNAQ*/*11*)^13^ are each found in ≈1% of aldosterone-producing adenomas (APAs). Exon-3 mutations of *CTNNB1* inducing constitutive activation of Wnt signaling are found in ≈5% of APAs,^14^ often coexisting with 1 of the above mutations. These mutations have also been reported in other tumors of the adrenal and other tissues. Whether these genes have a conserved role in aldosterone secretion or are particularly susceptible to mutation may be explored by investigating feline aldosterone-producing tumors. One route to hyperaldosteronism on which the cat may shed light is the role of leutinizing hormone (LH) and its receptor LHCGR (leutinizing hormone/chorionic gonadotrophin receptor). In humans, we reported a small group of patients with ≈100-fold increased expression of LHCGR due to the coexistence of somatic mutations in *CTNNB1* and the homologous G proteins, GNA11 or GNAQ (G protein subunit alpha 11 or Q).^13^ Since most domestic cats are neutered, upregulated LHCGR might be a commoner phenomenon.

Whole exome sequencing has identified novel candidate gene variants for polycystic kidney disease and frameshift mutations in rickets in cats,^15,16^ but kits for whole exome sequencing on cats are not commercially available. We, therefore, used whole genome sequencing (WGS) to identify somatic mutations in feline aldosterone-producing tumors. Additional tumors were screened using targeted Sanger sequencing for mutations in genes commonly mutated in human PA and for further instances of the somatic mutations identified with WGS. RNA sequencing was undertaken as an additional clue to similarities and differences between the regulation of feline and human aldosterone secretion. As in studies of somatic mutations in human APAs, a combination of genome wide sequencing and transcription analysis enables filtering of variants by those associated with high expression, and identification of associated distinct phenotypes (such as the high LHCGR causing the presentation of PA in early pregnancy).^12,13,17,18^ It is hypothesized that somatic mutations associated with excessive aldosterone secretion in cats are analogous to mutations found in humans, but WGS also provides the opportunity to explore novel mutations.

## Methods

### Data Availability

The data that support the findings of this study are available from the corresponding author upon reasonable request.

### Case Recruitment

Cats were recruited from referral centers in the United Kingdom and United States and from a first-opinion geriatric cat clinic in London. The Royal Veterinary College’s Ethics and Welfare Committee approved the collection and use of samples (URN 20131258E and URN 20212060-3). Nontumor adrenal tissue was selected retrospectively from cats that had adrenal tissue stored postmortem and were normotensive during life, with plasma aldosterone concentrations <300 pmol/L (reference intervals vary between laboratories, but this value is typically at or slightly higher than the upper limit of the reference interval, with most cats with documented hyperaldosteronism having considerably higher concentrations). PA was diagnosed by the attending veterinarian using the current standard for diagnosis, based on the presence of excessive aldosterone concentrations and otherwise unexplained repeatable hypokalemia in the presence of a unilateral adrenal mass. Normal electrolyte reference ranges depend on the sample type and analyzer used but are typically in the range of 149 to 158 mEq/L for sodium and 3.5 to 5.5 mEq/L for potassium. Plasma renin activity is not widely available for cats, so was not always measured. Adrenal gland enlargement was confirmed by abdominal ultrasonography, computed tomography, or postmortem. One PA case did not have an aldosterone measurement but was hypertensive and hypokalemic with a unilateral adrenal tumor and following adrenalectomy, both hypertension and hypokalemia resolved. Hypertension in cats is based on the measurement of systolic blood pressure over 160 mm Hg in conjunction with hypertensive retinal lesions (including hemorrhage, bullae, retinal detachment, and tortuous vessels) or on demonstration of systolic blood pressure over 160 mm Hg on >1 occasion.^19^ Some cases were treated with unilateral adrenalectomy, while other cases were managed medically.

### Tissue Collection

Where tissue was collected prospectively (at adrenalectomy or during postmortem examination), a section of the adrenal tumor was stored in RNAlater stabilization solution (R0901; Sigma-Aldrich), and the rest was fixed in 10% formalin. Tissue stored in RNAlater was kept at 4 °C for 24 to 48 hours before draining and storing at −80 °C. Formalin fixation was for a minimum of 48 hours before processing and paraffin embedding. Some cases were recruited retrospectively, where formalin-fixed paraffin-embedded blocks or snap-frozen tissue had been stored. For both prospectively and retrospectively collected samples, hematoxylin and eosin–stained sections from the formalin-fixed tissue were examined by board-certified veterinary pathologists to confirm that masses were of adrenocortical origin and to classify tumors as adenomas or adrenocortical carcinomas (ACCs). This was based on mitotic rate, capsular or vascular invasion, and the presence of distant metastasis. No published guidelines exist for differentiating benign and malignant lesions in cats, and metastasis is rarely seen with aldosterone-producing adrenal tumors; therefore, the biologic and endocrinologic activity of most feline tumors is similar to human APAs. Nontumor samples for DNA extraction were obtained from blood collected in EDTA tubes, adrenal tissue adjacent to the tumor, or from another organ (stored in RNAlater).

### Nucleic Acid Extraction

DNA was extracted using the PureLink genomic DNA mini kit (K182001; Invitrogen) and RNA was extracted using the PureLink RNA mini kit (12183018A; Invitrogen), following the manufacturers’ protocols. Where tissue stored in RNAlater was unavailable, DNA and RNA were extracted from formalin-fixed paraffin-embedded tissue. Fifteen-micrometer sections were cut under RNAase-free conditions and stored at 4 °C until extraction using the AllPrep DNA/RNA FFPE Kit (80234; Qiagen), following the manufacturer’s protocol. RNA was reverse transcribed to cDNA using a high-capacity RNA-to-cDNA kit (4387406; Applied Biosystems).

### Whole Genome Sequencing

Genomic DNA from cases with paired tumor and nontumor tissue (n=5) or tumor tissue alone (n=3) was sent to Novogene (Beijing, China) for WGS. Samples underwent gel electrophoresis to check for DNA degradation and contamination, and DNA concentration was measured using the Qubit DNA Assay Kit in Qubit 2.0 Fluorometer (Life Technologies). DNA was randomly fragmented to an average size of 350 bp. DNA fragments were end polished, A tailed, and ligated with adapters of Illumina sequencing and further polymerase chain reaction (PCR) enriched with primers of P5 and P7 oligonucleotides. The PCR products were purified (AMPure XP system), followed by size distribution evaluation by Agilent 2100 Bioanalyzer (Agilent Technologies) and molarity measurement using real-time PCR. The Illumina NovaSeq 6000 platform (Illumina, Inc) was used to generate 150 bp paired-end reads to yield 81 Gb of data per sample. Sequencing depths over 24× were achieved for each sample that underwent WGS, and coverage was at least 97.4% with a depth of at least 4×.

### WGS Data Processing

Image data were converted into Fastq files via base calling using the Illumina pipeline consensus assessment of sequence and variation (CASAVA), version 1.8.2. Unusable reads were removed by Fastp, version 0.20.0, with parameters -g -q 5 -u 50 -n 15 -l 150 --min_trim_length 10 --overlap_diff_limit 1 --overlap_diff_percent_limit 10.^20^ Reads were aligned to the reference genome felis_catus_9.0 (GCA_000181335.4) using Burrows-Wheeler Aligner, version 0.7.7-r455, with default parameters.^21^ Duplicate removal was performed using Samtools, version 1.3.1. BAM files for samples were merged using Picard, version 1.111 (http://picard.sourceforge.net). Genome analysis toolkit (GATK), version 3.5,^22^ with the parameter: -T MuTect2 detected somatic single-nucleotide polymorphisms (SNPs) for samples with paired tumor and nontumor DNA available. Where only tumor DNA was available, SNPs were called using Samtools, version 1.3.1, with parameters -C 50 – m 2 -F 0.002 -d 1000. Strelka was used to call somatic small insertions and deletions (InDels), and Control-free copy number caller was used to call somatic copy number variants (CNVs), which were filtered to include only exonic variants. Annotate variation (ANNOVAR), version 2015Dec14, was used to annotate variants. Ensembl variant effect predictor^23^ was used to predict the effect of SNPs. Data were filtered to select SNPs and InDels that passed quality control, remove any unannotated or noncanonical variants, and select missense SNPs predicted as deleterious by sorting intolerent from tolerent (SIFT) with a threshold of SIFT ≤0.05.^24^ Genes of interest were selected for confirmation with targeted sequencing on the basis of known high expression in human aldosterone-secreting tissue using genotype-tissue expression data^25^ or because of a suspected role in aldosterone secretion. PolyPhen2 HumVar scores are reported for genes of interest. Canonical protein sequences for each gene from cats and humans were aligned using the National Centre for Biotechnology Information's basic local alignment search tool (BLAST) and UniPro UGENE; analogous amino acids were identified in human proteins.^26,27^ The catalogue of somatic mutations in cancer (COSMIC; cancer.sanger.ac.uk)^28^ was used to explore whether analogous somatic mutations have been identified in human tumors.

### Targeted Sequencing

Mutations identified by WGS were selected for confirmation with bidirectional targeted sequencing. Genes were selected if analogous mutations arise in human APAs (*CTNNB1*, *GNAQ*, *CACNA1C*, and *ARHGAP12*), for genes with a known role in steroidogenesis (*CYP21A2*),^29^ or genes with a potential role in calcium-dependent aldosterone secretion (*PCDH15*). Primers were designed using Primer-BLAST^30^ to target regions of interest from genes identified by WGS in the present study and genes commonly mutated in human PA tumors (*KCNJ5*, *ATP1A1*, and *ATP2B3*). PCRs were performed on 200 ng DNA using AmpliTaqGold Fast PCR Master Mix (4390939; Thermo Fisher Scientific) in a 20-μL reaction in a thermocycler for 35 cycles (Table S1). For touchdown reactions, there was a sequential reduction in annealing temperature from 68 °C to 58 °C in 1 °C steps over 10 cycles, followed by 25 cycles at 57 °C. ExoSAP-IT PCR product cleanup reagent (78200; Thermo Fisher Scientific) was used to enzymatically hydrolyze excess primers and nucleotides in the PCR product. Cleaned PCR products were mixed with primers and sequenced in both directions using the LightRun tube barcode Sanger sequencing service (Eurofins Genomics, Germany). UniPro UGENE was used to align sequenced amplicons to the reference sequence. 4peaks was used to generate figures from sequencing files.

### RNA Sequencing

RNA sequencing was performed on PA (n=5) and control cases (n=4); control cats were all female neutered domestic shorthairs aged 14.9 to 17.7 years, with plasma aldosterone concentrations (58–146 pmol/L) below the upper end of the assay reference interval (388 pmol/L). RNA samples were submitted in 2 batches to the Genome Centre (Blizard Institute, Queen Mary University of London) for library preparation and sequencing. Samples were analyzed on an Agilent 2100 Bioanalyzer to calculate RNA integrity number, and Nanodrop analysis was performed to check concentration and purity. Globin mRNA and rRNA were depleted using the NEBNext Globin mRNA and rRNA Depletion Kit (E7755L/X; New England Biolabs), whereby DNA probes targeting unwanted RNAs were hybridized to RNA and digested with RNase H, followed by cleanup with DNase I and RNA sample purification beads. Directional RNA library preparation was performed using the NEBNext Ultra II Directional RNA Library Preparation Kit for Illumina (New England Biolabs; E7760S/L) and NEBNext Multiplex Oligos for Illumina (E6609S; New England Biolabs). RNA was fragmented and primed, then converted to cDNA and purified using AMPureXP beads (A63881; Beckman Coulter). NEBNext adapters were ligated and then the ligation reaction was purified using AMPure XP beads targeting insert sizes of 300 bp. This was followed by PCR enrichment of the library and purification with AMPure XP beads. The Qubit 2.0 Fluorometer was used to assess library size and molarity. Libraries were diluted, pooled, and cleaned using AMPur eXP beads. Size and molarity were analyzed on the Qubit 2.0 Fluorometer and Agilent 2200 Bioanalyzer. Pooled libraries were denatured and diluted to 20 pmol/L before paired-end sequencing on the Illumina NextSeq to generate >11.9 million 150 bp reads per sample.

### RNA Sequencing Data Processing

Fastq files from each sampling lane, included samples sequenced in both batches, were merged to generate a single merged Fastq file for each sample. FastQC (Babraham Bioinformatics) was performed on a subset of the data. Spliced Transcripts Alignment to a Reference aligner was used to align reads to the felis_catus_9.0 genome. R, version 4.2.2 (october 31, 2022 Innocent and Trusting), was used to extract count matrices. Batch correction was performed using ComBat-seq within the sva package (version 3.46.0). Sample 9 was treated as batch 2 despite sequencing in both batches, as it was most similar to this batch and batches must be >1. Normalized count files were generated using DESeq2 (version 1.38.1). Prism 10 (version 10.0.0) was used to visualize normalized gene counts. Rows with a sum count under 50 were removed from the batch corrected data. Differential gene expression analysis using DESeq2 compared normal adrenal to tumor and adenoma to ACC transcriptomes. Significance was set at an adjusted *P*<0.05.

## Results

### Cats With Aldosterone-Secreting Adrenal Tumors

Adrenal tumors from 13 cats with PA were recruited, 9 from adrenalectomy and 4 collected at postmortem examination (Table S2). Cats with PA were aged 7 to 18 years. All PA cats were neutered; 7 were males and 6 females, and all were domestic crossbreeds. Pretreatment systolic blood pressure for cats with hypertension (10/11) ranged from 165 to >250 mm Hg. The cat that was not deemed hypertensive had a systolic blood pressure of 158 mm Hg, and 1 cat was not amenable to blood pressure measurement. Plasma aldosterone concentrations of PA cases ranged from 328 to >5000 pmol/L. Tumors were classified as adenomas (n=5) or ACCs (n=8), based on the presence of gross or microscopic local invasion (n=6), distant metastasis (n=1), or mitotic rate above 3 per 10 high-powered fields (n=5).

### Somatic Mutations Identified by WGS and Sanger Sequencing

Before filtering based on SIFT ≤0.05, the adenoma (n=1) had 24 and the ACCs (n=4) had a median of 40 (range, 25–55) somatic missense SNPs. Sixty-seven somatic missense SNPs within canonical sequences with predicted significant effect on protein function (SIFT score ≤0.05) were identified within the 5 paired samples (Table S3); the adenoma had 6, while the ACCs had a median of 18 (range, 8–20; n=4) somatic missense SNPs. No somatic SNPs were found at identical residues in >1 sample, and none were present in cases without paired normal tissue (Table 1). Forty-three CNVs and 1 InDel were detected in the adenoma case, while 123 to 610 CNVs and 4 to 6 InDels were detected in the ACCs (n=4; Table S4). Twenty identical CNVs were present in >1 sample (Table S5), but no identical InDels were present in >1 sample.

**Table 1.**
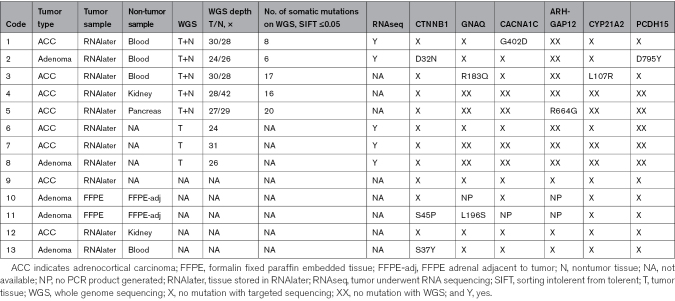
WGS Sample Information, Sequencing Depth, and Somatic Mutations Identified

**Figure 1.**
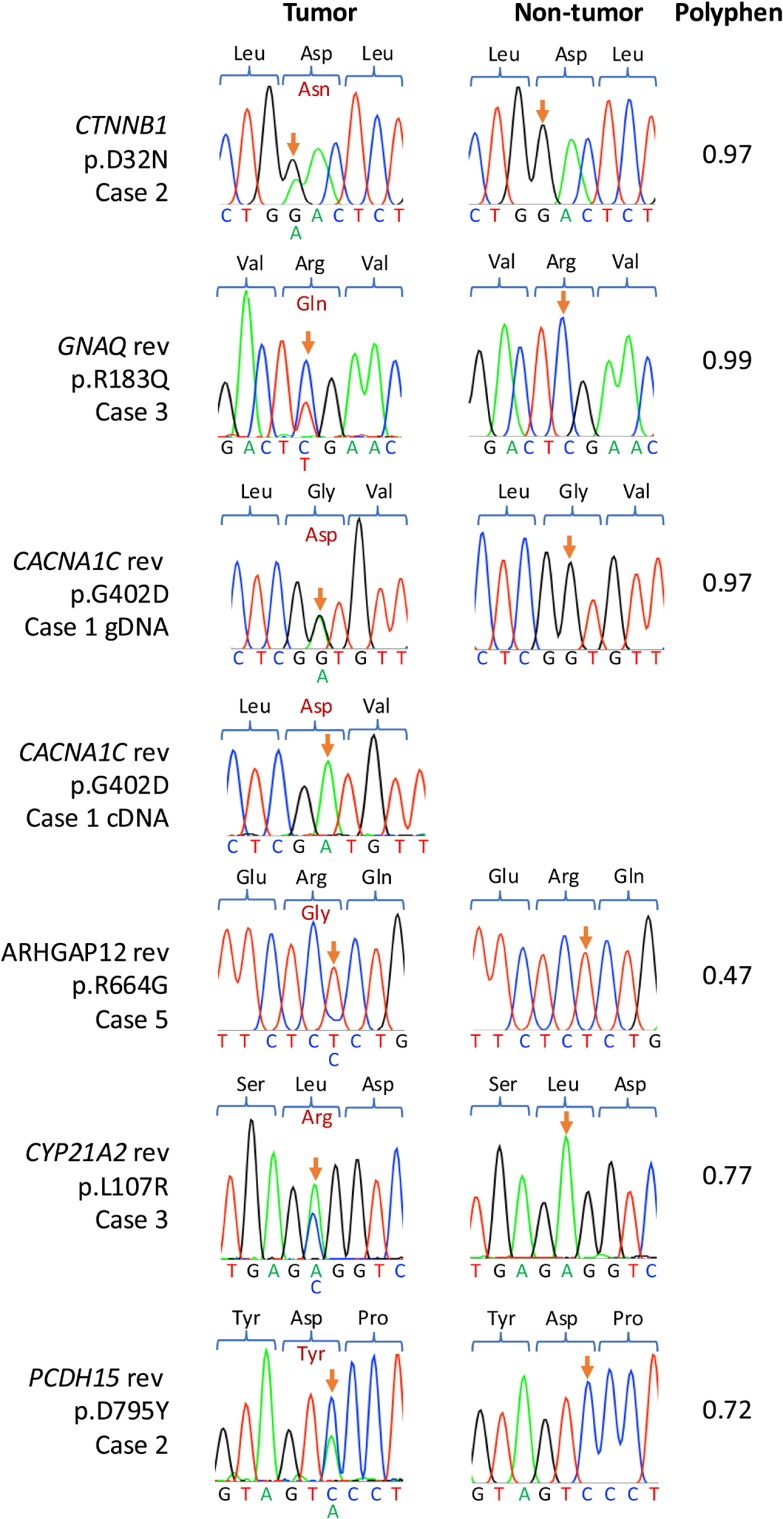
**Sanger sequencing of the somatic mutations of interest identified by whole genome sequencing and associated PolyPhen2 scores.** Orange arrows highlight relevant residues in each sequence. Somatic mutations are confirmed in case 2 (*CTNNB1* p.D32N), case 3 *GNAQ* p.R183Q reverse, case 1 *CACNA1C* p.G402D reverse, *ARHGAP12* p.R664G reverse, *CYP21A2* p.L107R reverse, and *PCDH15* p.D795Y reverse for tumor and nontumor, respectively. The sequence for cDNA is shown for *CACNA1C* as the residue was observed to be homozygous for the alternate allele on RNA sequencing. Arg indicates arginine; Asn, asparagine; Asp, aspartic acid; gDNA, genomic DNA; Gln, glutamine; Gly, glycine; Leu, leucine; Pro, proline; Rev, reverse; Ser, serine; Tyr, tyrosine; and Val, valine.

Targeted Sanger sequencing confirmed the following somatic mutations identified on WGS: *CTNNB1* p.D32N, *GNAQ* p.R183Q, *CACNA1C* p.G402D, *ARHGAP12* p.R664G, *CYP21A2* p.L107R, and *PCDH15* p.D795Y (Figure 1). All somatic mutations were confirmed as heterozygous within the tumor and absent within control tissue. The *CACNA1C* mutation was heterozygous on WGS and targeted sequencing of genomic DNA but homozygous for the alternate allele on targeted Sanger sequencing of cDNA and RNA sequencing.

**Figure 2.**
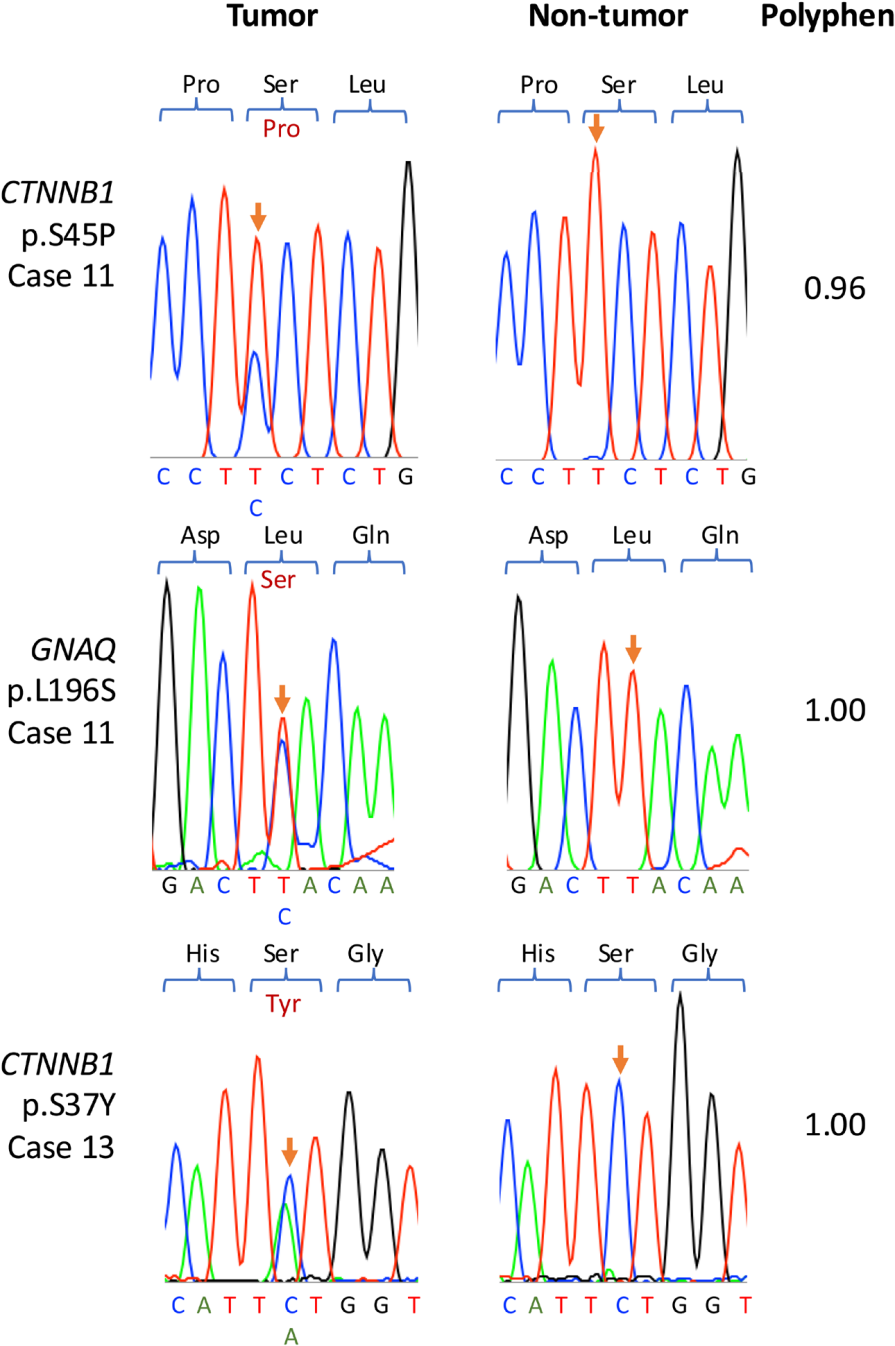
**Sanger sequencing of samples that did not undergo whole genome sequencing (WGS), and additional mutations were identified with associated PolyPhen2 scores.** Orange arrows highlight relevant residues in each sequence. Genomic DNA was used to sequence *CTNNB1* in case 11 and case 12, and cDNA was used to sequence *GNAQ* in case 11 for tumor and nontumor samples.

**Figure 3.**
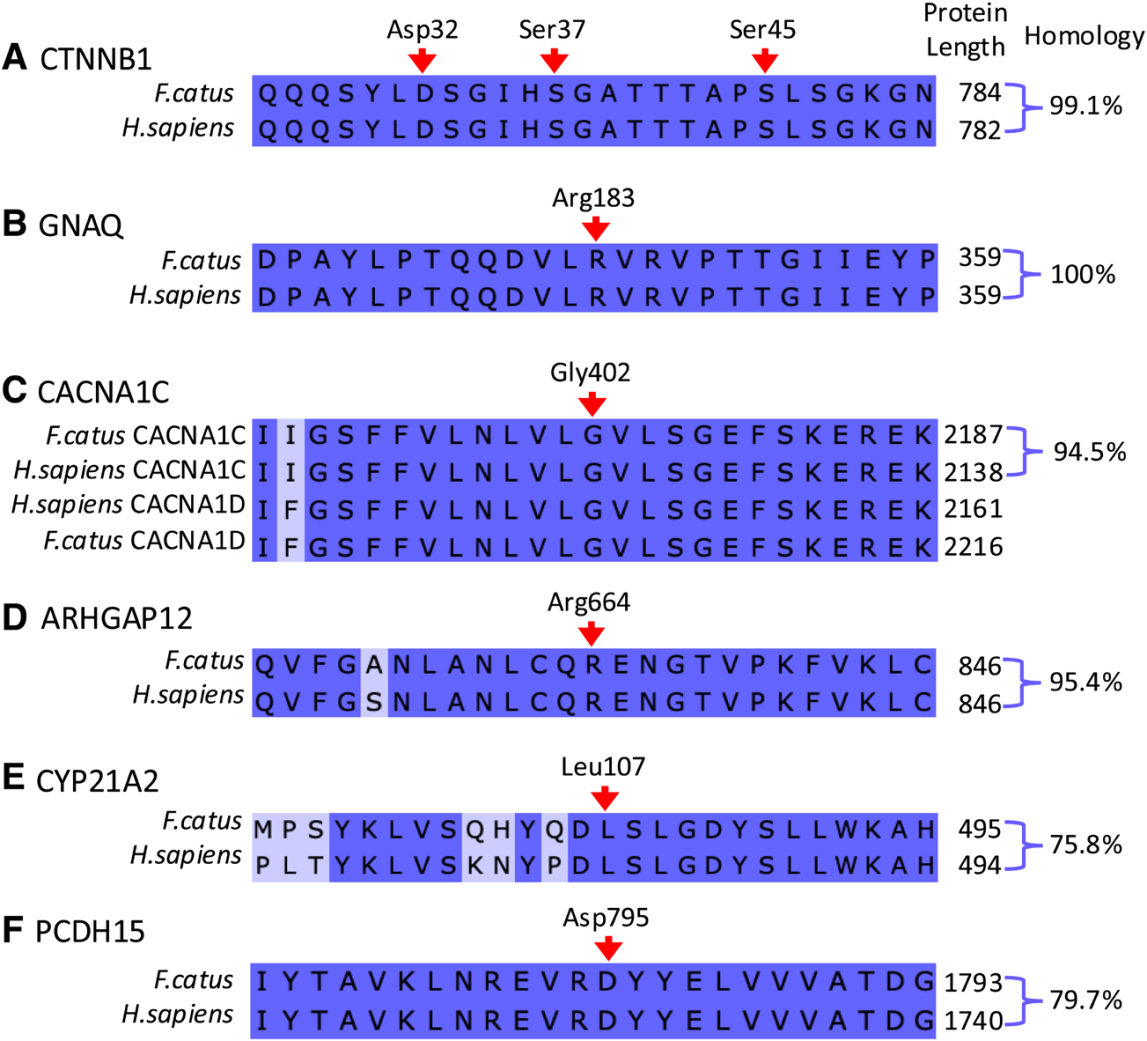
**Alignment of human and cat protein sequences for mutated genes of interest.** CTNNB1 (Canonical protein sequences for catenin beta 1; **A**), GNAQ (G protein subunit alpha Q; **B**), CACNA1C (calcium voltage-gated channel subunit alpha1 C; **C**), ARHGAP12 (rho GTPase activating protein 12; **D**), CYP21A2 (cytochrome P450 family 21 subfamily A member 2; **E**), and PCDH15 (protocadherin related 15; **F**) for *Felis catus* (*Fcatus*) and *Homo sapiens* (*H* *sapiens*). Conserved residues are dark purple, and residues that differ between the aligned proteins are lilac. Protein lengths and sequence homologies are given to the right of the alignments. Somatic mutation sites are indicated by a red arrow, and each mutated site is conserved between cats and humans.

Three somatic mutations were identified using targeted Sanger sequencing at residues in the same genes, but distinct from the amino acid residues identified by WGS (Figure 2), including 2 further *CTNNB1* mutations at p.S45P and p.S37Y in cases 11 and 13, respectively, both of which were in adenomas. In case 13, alongside *CTNNB1* p.S45P, a somatic *GNAQ* p.L196S mutation was identified. There was over 94% homology between cat and human protein sequences for all genes of interest except CYP21A2 and PCDH15 (75.8% and 79.7% homology, respectively), and the mutated residue and surrounding sequences were well conserved between species (Figure 3). *GNAQ* shares 100% amino acid sequence homology with humans; however, no *GNAQ* p.L196 substitutions are reported on COSMIC (cancer.sanger.ac.uk).^28^ No mutations were identified in *KCNJ5*, *CACNA1D*, *ATP1A1*, or *ATP2B3*, the genes most frequently affected in humans with PA.

### RNA Sequencing: Gene Expression

**Figure 4.**
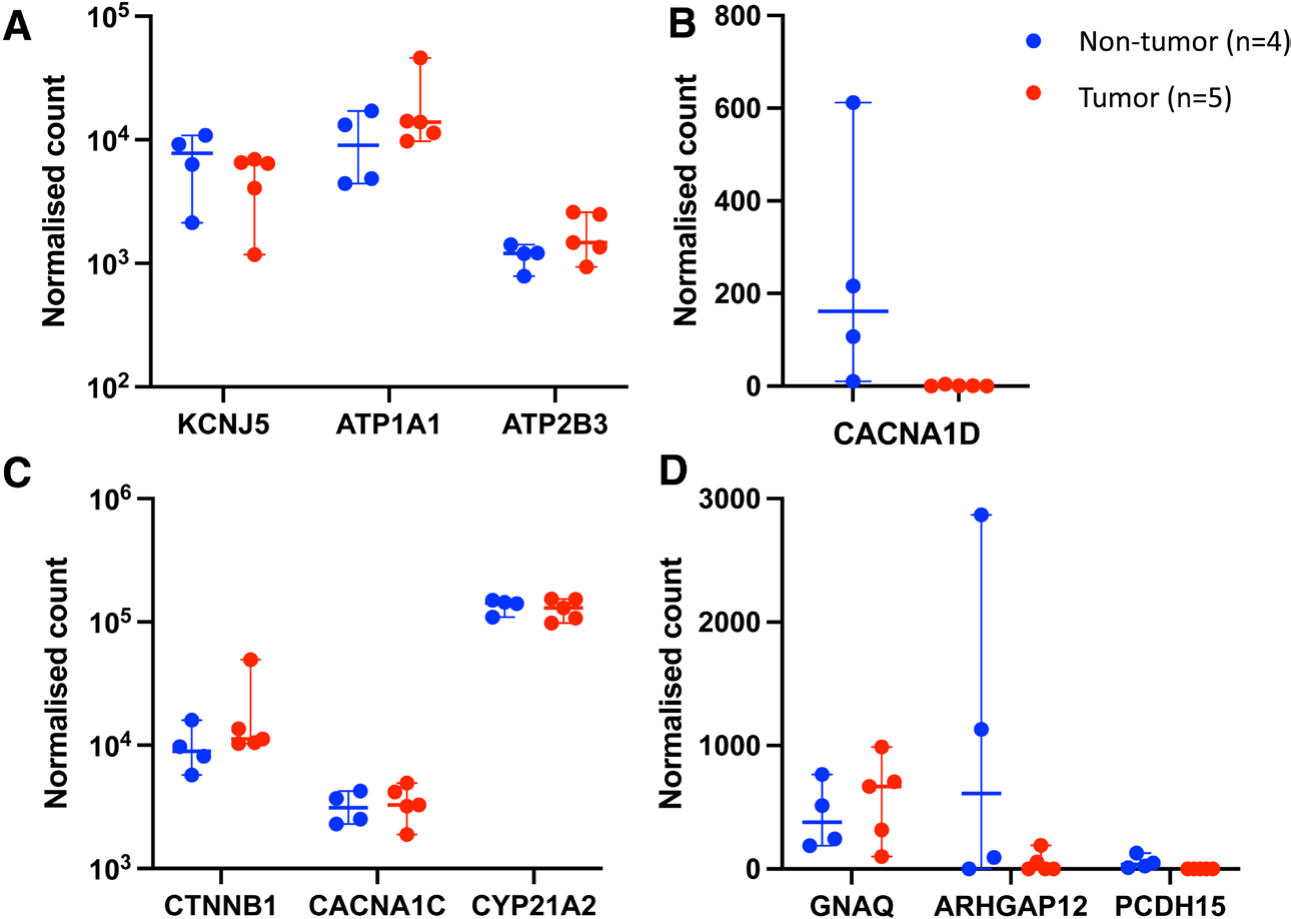
**Normalized gene counts from RNA sequencing of nontumorous adrenals from cats without hyperaldosteronism (n=4, blue) and feline aldosterone-secreting adrenal tumors (n=5, red).** Genes commonly mutated in human aldosterone-producing adenomas (**top**) were split into those with higher expression (**A**, logarithmic *y* axis) and lower expression (**B**, linear *y* axis). Expression of genes with somatic mutations identified in feline aldosterone-producing tumors (**bottom**) is shown with higher expression (**C**, logarithmic *y* axis) and lower expression (**D**, linear *y* axis). Horizontal bars display median expression, and whiskers give the 95% CI.

RNA sequencing was used to explore gene expression within 4 control adrenal glands and 5 aldosterone-producing tumors. Genes commonly mutated in humans, including *KCNJ5*, *ATP1A1*, and *ATP2B3*, were expressed at medium to high levels in feline adrenal tissue (Figure 4A), while *CACNA1D* expression was low in both nontumorous adrenals and aldosterone-secreting tumors (Figure 4B). The expression of genes with somatic mutations identified in feline aldosterone-producing tumors was also explored. Expression of genes, including *CTNNB1*, *CYP21A2*, and *CACNA1C*, was high (Figure 4C); *GNAQ* expression was moderate, and *PCDH15* and *ARHGAP12* expression was low (Figure 4D). *CACNA1C* was the second most highly expressed calcium channel subunit in feline adrenal tissue (Table 2). *CACNA1D* expression was lower (*P*<0.001) in tumor than in nontumor tissues. There were no reads for LHCGR in tumor or control adrenals, despite the likely inclusion of the zona reticularis from the nontumorous adrenals. There was no clear clustering of tumors classified as adenomas compared with carcinomas (Figure S1A), although 595 genes were differentially expressed (Figure S1B).

**Table 2.**
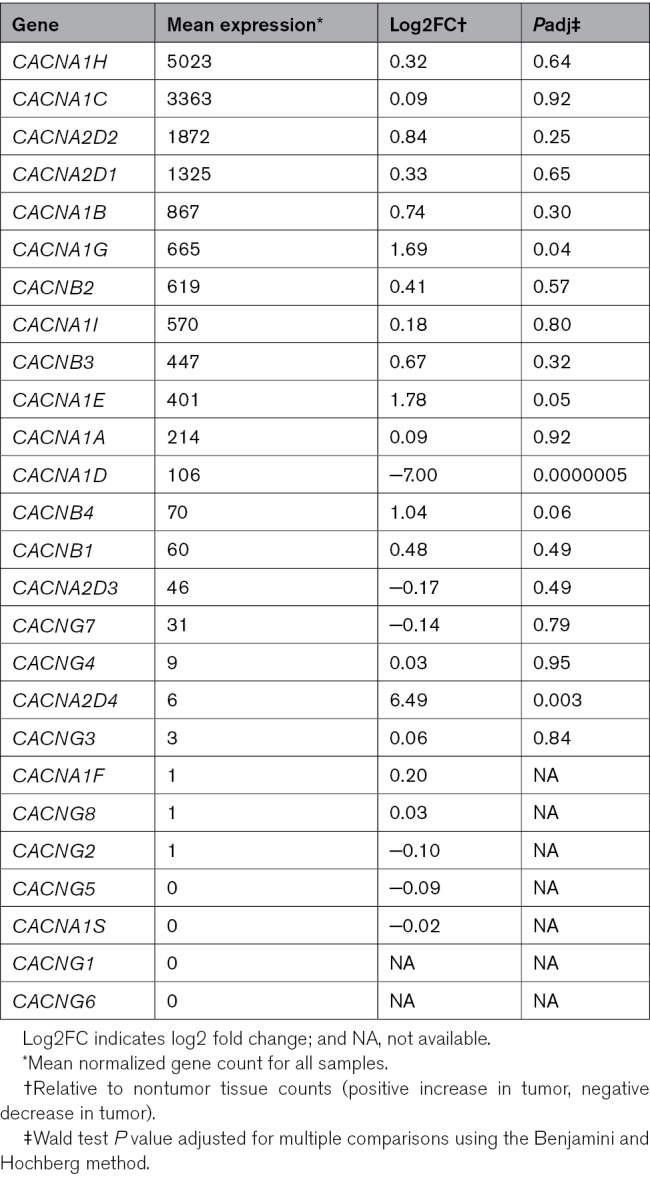
Mean Normalized Gene Count for Calcium Channels from Deseq2 Analysis of RNA Sequencing Data of Feline Nontumorous Adrenal Tissue (n=4) and Aldosterone-Secreting Tumors (n=5)

## Discussion

A mix of genome-wide and targeted sequencing has discovered multiple somatic mutations associated with feline hyperaldosteronism. There were more similarities to, than differences from, the somatic mutations found in human PA. Several predicted deleterious mutations in *CTNNB1* and *GNAQ* were identified. A further similarity between the species was a mutation identified in *CACNA1C* at the analogous residue to that identified in *CACNA1D* in humans.^17,31^ Its significance was enhanced by our parallel finding, on transcriptome analysis, that the relative abundance of *CACNA1C* and *CACNA1D* in the adrenal is reversed between humans and cats, the latter expressing almost no *CACNA1D*. No mutations were identified in other genes, *KCNJ5*, *CACNA1D*, *ATP1A1*, or *ATP2B3,* in which somatic mutations are frequently identified in human APAs, nor in *GNA11*, *CADM1*, or *SLC30A1.* No recurrent mutations were found in other genes, but single novel examples of predicted deleterious mutations were identified in genes not reported previously in human APAs.

Three mutations of *CTNNB1* were identified: p.D32N, p.S37Y, and p.S45P, all residing within exon 2 in the cats, equivalent to exon 3 in humans. Phosphorylation of several residues here is required to prevent the encoded β-catenin, in the Wnt system, from entering the nucleus and redirecting cell differentiation. Multiple somatic mutations have been identified in human APAs (≈5%),^14^ ACCs (≈16%, including the S45P mutation in the ACC from which H295R cells were derived),^32^ and other cancers.^33^ Somatic *CTNNB1* p.D32N and p.S37Y mutations have not been described in human APAs but have been described in other human tumors. All 3 feline tumors harboring *CTNNB1* mutations were classified as adenomas. It remains uncertain whether *CTNNB1* mutations cause autonomous aldosterone production or tumor-like behavior of the APAs.^34^

*GNAQ* encodes a guanine nucleotide-binding protein subunit-α, Gaq, which mediates the physiological aldosterone response to angiotensin II.^13^ Somatic *GNAQ* p.R183Q and p.L196S mutations were identified in feline tumors in this study. The amino acid sequence for GNAQ is 100% conserved between humans and cats. *GNAQ* p.R183Q mutations cause uveal melanomas and vascular malformations (including Sturge-Weber syndrome) in humans,^35,36^ although these are observed less frequently than a mutation at p.Q209, which is also the sole site reported (to date) in human APAs.^13^ The p.R183 and p.Q209 residues of GNAQ are analogous to p.R201 and p.Q227 of GNAS (guanine Nucleotide binding protein), where mutations similarly fix Guanosine triphosphate (GTP) in a nonhydrolysable position and cause constitutive cAMP activation in McCune Albright Syndrome.^37^,^38^ It has been a puzzle why the p.Q209 mutations of GNA11/Q causing constitutive activation of Gα11 or Gαq (the encoded G proteins) are not alone sufficient to cause autonomous aldosterone production, considering that these G proteins mediate the aldosterone response to angiotensin II in the adrenal.^13^ So our discovery here of a tumor with a known gain-of-function mutation of GNAQ, but no mutation of CTNNB1, may anticipate the eventual discovery of human patients with similarly isolated mutations of GNA11 or GNAQ. Conversely, the coexistence of our novel GNAQ mutation at p.L196 with the well-known CTNNB1 p.S45P mutation shows that the processes driving the double mutation are not limited to the human adrenal or to the p.Q209 residue. GNAQ p.L196 is part of the highly conserved tetrapeptide-β2 sheet of the encoded Gα_11__/q_, and the p.L196A substitution reduces the affinity of the G proteins for the calcium receptor.^39^ Since leucine and alanine are similar amino acids with hydrophobic side chains, substituting with a more dissimilar amino acid, such as serine, an amino acid with a polar uncharged side chain, is highly likely to have functional consequences. While we cannot be 100% certain that the *GNAQ* p.L196S mutation is functional, we suspect this from its coexistence with the *CTNNB*1 mutation and from previous analysis of homologous mutations of GNA11.^39^

Although somatic *CACNA1C* mutations have not been identified in humans with PA, gain-of-function mutations in the homologous genes *CACNA1D* and *CACNA1H* have been identified, and *CACNA1D* is the commonest genotype of APAs in Black patients and in aldosterone-producing micronodules.^9,40^ Amino acid sequence identity between CACNA1C and CACNA1D proteins is 75% in humans and 69% in cats.^41,42^ Mutations in calcium channels cause a variety of well-studied changes to membrane calcium currents that increase intracellular calcium and, therefore, aldosterone secretion.^31,43^ Somatic *CACNA1D* p.G403R mutations slow the voltage-dependent inactivation of channels,^17^ and analogous *CACNA1C* p.G402S mutations causing Timothy syndrome cause similar effects.^17,44^ In normal human adrenals, *CACNA1C* expression is confined to vascular cells; however, inspection of recent single-cell data from humans shows that *CACNA1C* is coexpressed in steroidogenic cells in APAs.^45^ Analysis of calcium channel expression and identification of a somatic mutation with suspected functional effects in the feline adrenal tissue suggest that *CACNA1C* may have a role in cats analogous to that of *CACNA1D* in humans. The *CACNA1C* mutation was heterozygous at the genomic DNA level, but only the alternate allele was expressed at the RNA level (on RNA sequencing and targeted sequencing of cDNA), indicating sole expression of the mutated voltage-dependent L-type calcium channel subunit-α protein. The reason for reduced expression of the wild-type gene is unclear. Interestingly, the reverse has been reported for *GNA11* p.Q209 mutations in adrenal tissue adjacent to human APAs, where cDNA was heterozygous but genomic DNA was homozygous.^13^ Selective expression of *CACNA1C* in cat tumors may explain the apparently greater effectiveness of the nonselective dihydropyridine calcium channel blocker amlodipine in the treatment of hypertension due to PA in cats, when compared with humans.^46–49^

Three mutations were identified in 1 sample each and were selected to be of interest due to the identification of a previous similar mutation in a single human APA in *ARHGAP9* (*ARHGAP12*),^50^ their role in steroidogenesis (*CYP21A2*),^29^ or *PCDH15*.^25^ No additional instances of these mutations were identified with targeted sequencing in this study. If further mutations are identified in these genes in the future, in either cat or human samples, it would be interesting to explore the effects of these mutations on aldosterone secretion. The absence of mutations in *KCNJ5*, *ATP1A1*, and *ATP2B3* in cat tumors may reflect differences in pathway importance in cats, or their absence may simply be due to the sample size. Similar sized studies of human APAs have missed common mutations.^17,51^

The pathogenesis of feline PA is likely to be heterogeneous, as in humans, where tumors of different somatic genotypes may have different origins,^45^ and vary markedly in relative prevalence among patients of different ethnicities.^8,40,52–54^ A higher proportion of feline adrenal tumors are described as ACCs than human adrenal tumors; human ACCs are rare and less likely to secrete aldosterone. The finding that 4 of 13 cats had the same or analogous mutation to those reported in humans; therefore, seems remarkable and suggests a common pathogenesis of aldosterone-producing tumors in both species. Domestic cat foods, like typical human diets, have high salt content. However, the results reported here do not directly support or refute the maladaptive response to salt.^6,55^ It may be that the Wnt system, as well as being critical to adrenocortical development, drives appropriate downregulation of aldosterone when not required but the opposite when activated. Although only a minority of APAs have *CTNNB1* mutations, zona glomerulosa tissue adjacent to most APAs shows features of Wnt activation.^56^

An alternative hypothesis for the driver of tumorigenesis in cats is the neutered status of many domestic cats, leading to high circulating levels of LH.^57^ The absence of LHCGR expression by RNA sequencing of tumorous and normal adrenal tissue probably disproves the LH hypothesis. It also contrasts with human APAs in which higher LHCGR expression is found compared with normal adrenals,^58^ especially in tumors with both *GNAQ* and *CTNNB1* mutations.^13^

The somatic SNP load per feline ACC was slightly higher than the mutation load reported in human ACCs (median, 17; range, 1–806); however, it should be noted that the range in human ACCs was large.^32^ Fewer InDels and CNVs were identified in adenomas than in the ACCs; comparable numbers of CNVs are present in human adrenocortical tumors.^59^ Several CNVs were identified within >1 tumor and affected regions containing genes (including CACNA1H and SLC24A2), which are of interest in human PA.^9,60^

The study has limitations; the number of feline tumors was smaller than in recent whole exome sequencing and RNA sequencing of human APAs.^12,13,18^ It is, however, comparable to older studies, including the landmark discovery of KCNJ5 mutations.^17,31,50,51^ In the present study, it was assumed that the whole tumor was secreting aldosterone. In human APAs, aldosterone synthase (CYP11B2) expression is not homogenous, and a higher sensitivity for detecting mutations can be achieved by selecting DNA or RNA from CYP11B2-positive areas.^61^ As cats have a single *CYP11B* gene, it is not possible to select for aldosterone-producing feline tissue by immunohistochemical staining for aldosterone synthase. In both the tumors and normal adrenals, bulk RNA sequencing represents the average of gene expression across the sample of tissue analyzed, and samples may contain variable proportions of each layer of the adrenal gland. In some cats, the diagnosis of primary hyperaldosteronism was made in the absence of renin measurements. Renin measurement is not widely available clinically for cats; therefore, the criteria of elevated plasma aldosterone concentrations in the presence of otherwise unexplained hypokalemia and an adrenal mass on imaging were used, which is the current diagnostic standard for cats.^62,63^ It is of interest to us that in our recent prospective study of PA in humans,^18^ only 3 of 170 patients had a plasma Na^+^ <140 mmol/L. The range of plasma Na^+^ in cats is considerably higher, but we note that all the cats had a plasma Na^+^ ≥150 mmol/L (Table S2). Finally, we have not tested whether the predicted deleterious effect of the novel gene mutations we found stimulates aldosterone production. In the absence of high transcription rates in aldosterone-secreting tissue and evidence of recurrence (several occurrences of the same or similar mutations in a gene), it has not been our practice to continue to the transfection of wild type and mutant experiments required to interrogate the functional consequences of a novel mutation. This study did not illuminate new pathways of aldosterone regulation, as we identified previously unreported genes in the absence of recurrent somatic mutations.

## Perspectives

In conclusion, a mixture of whole genome and targeted sequencing found that cats, a nonhuman species that develops spontaneous aldosterone-producing tumors, share several of the mutated pathways that cause human PA. In all but 1 case (*CTNNB1* p.S45P mutation), the exact amino acid residue or gene is subtly different from the human counterpart. This finding is consistent with our hypothesis that cats and humans have common underlying drivers of mutation with consequences that vary with the exact sequence of DNA and its rate of transcription.

## ARTICLE INFORMATION

### Acknowledgments

The authors thank James Boot, Nadiya Mahmud, Eva Wozniak, and Charles Mein for their assistance with RNA sequencing; Norelene Harrington for help classifying tumors; and those who contributed cases from the United Kingdom (including the Queen Mother Hospital, the Ralph Veterinary Referral Center, Southfields Veterinary Specialists, and Dovecote Veterinary Hospital) and the United States (including Cummings School of Veterinary Medicine at Tufts University, Pieper Veterinary, and Rose City Veterinary Hospital).

### Author Contributions

A. Watson, H. Syme, and M. Brown designed the study. A. Watson collected and submitted samples, performed data analysis, and wrote the first draft of the article. All authors contributed to the writing of the article. A. Watson and M. Brown had full access to the data in the study.

### Sources of Funding

A. Watson holds a London Interdisciplinary Doctoral Training Program PhD studentship funded by the Biotechnology and Biological Sciences Research Council (BB/T008709/1) and Boehringer Ingelheim.

### Disclosures

None.

### Supplemental Material

Tables S1–S5

Figure S1

## Supplementary Material


