# Supplementary material for: Somatic GNAQ, CTNNB1, and CACNA1C Mutations in Cat Aldosterone-Secreting Tumors
Source: Hypertension. 2024 Oct 21;81(12):2489–500. doi: 10.1161/HYPERTENSIONAHA.124.23501 (PMC11578054; doi:10.1161/HYPERTENSIONAHA.124.23501)

**Online-only supplement**

# Somatic GNAQ, CTNNB1 and CACNA1C mutations in cat aldosterone secreting tumors

Alice Watson MA VetMB MRCVS, Royal Veterinary College, London

Harriet Syme BSc BVetMed PhD DipACVIM DipECVIM-ca FHEA MRCVS, Royal Veterinary College, London

Morris Brown, MD FRCP FMedSci, Queen Mary University of London, London

**Short title**: Somatic mutations in cat adrenal tumors

**Corresponding author**: Morris Brown, morris.brown@qmul.ac.uk, Department of Clinical Pharmacology, William Harvey Research Institute, Queen Mary University London, London, United Kingdom

**Supplementary Table S1.** Primers used for targeted Sanger sequencing. Size; PCR product length, Tm; Annealing temperature (or touchdown protocol), cDNA; complementary DNA, gDNA; genomic DNA.

| Gene | Forward | Reverse | Size | Tm | cDNA/gDNA | Exon |
|---|---|---|---|---|---|---|
| **KCNJ5 p.151-168** | CACTTGGCTGTTCTTCGGTT | TCTGCTCTCTTCTTTGGCTGA | 297 | 62 | c/g | 2 |
| **ATP1A1 p.104** | AGCTGCTGAGATCCTGGC | ACTTTGTGCACACTTTGTCATC | 223 | 60.5 | g | 4 |
| **ATP1A1 p.104** | | AGGACCACGCCAAGATACAG | 207 | 60 | c | 4-5 |
| **ATP1A1 p.960** | GCATGGATGGCTTTGGAAGA | AGGAGCGACTTACTTGAGGG | 183 | 62 | g | 21 |
| **ATP1A1 p.960** | GCCTTCTTCGTCAGCATCG | AGTAGGGGAAGGCACAGAAC | 223 | 60 | c | 21 |
| **APT1A1 p.332** | ATCCACATCATCACGGGTGT | AGGAGTCTCACATGCAAGCT | 208 | 62 | g | 8 |
| **APT1A1 p.332** | | GGTCTCCACGGCTTCTAAGT | 225 | 60 | c | 8-9 |
| **ATP2B3 p.425** | CGTCATCATCCTGGTCCTCT | AAGGCATTCTCACACGTACC | 230 | 60.5 | g | 12 |
| **ATP2B3 p.425** | TCACCGTCATCATCCTGGTC | ACCAGGTTGTTGTCCCTCAT | 223 | 60 | c | 12 |
| **ATP2B3 p.1107** | GGGAGGAAGAGATCGACCAT | CCTGGGCTCTGACGGAAG | 222 | 62 | g | 23 |
| **ATP2B3 p.1107** | CGGGAAAGGACGAGATGACC | CACCCTGAAATGAACCGCTT | 171 | 62 | c | 23-24 |
| **GNA11 p.209** | AGATGTCTGTCTGCGGGC | TTGGTCGTACTCACTCAGGG | 162 | 59 | g | 5 |
| **CTNNB1 p.32** | CTGATGGAACTGGACATGGC | TGTTCCCACTCGTACAGGAC | 185 | 60.5 | c/g | 2 |
| **PCDH15 p.795** | TTTCGCATCACATCCAACGG | GGCTGGCAGGTTCTCTTCTA | 216 | 59 | c/g | 21 |
| **CYP21A2 p.107** | CTGTGGCTTCCTTGGTCAGT | GCCTCACCTCACAGAACTCC | 204 | Touchdown | g | 3 |
| **CYP21A2 p.107** | ATGGGTAGACTTTGCTGGCA | CTCATGCGCTCACAGAACTC | 224 | Touchdown | c | 2-3 |
| **CACNA1C p.402** | ACTGGGTCAATGATGCCGTA | CCCTTCAGGTCCTCTTCCAG | 199 | Touchdown | c | 8 |
| **CACNA1C p.402** | GTGCCTCACTAACTATCATTCCA | GGTGGCCTCTTATCCCAGG | 249 | Touchdown | g | 8 |
| **GNAQ p.183** | AATGACTTGGACCGTGTTGC | CTTCTCTCTGACCTCTGGCC | 158 | Touchdown | c | 4 to 5 |
| **GNAQ p.183** | TTGTGTCTTCCCTCCTCCAG | GCTGGAAGGTGGGTTTTATGG | 183 | Touchdown | g | 4 |
| **ARHGAP12 p.664** | GAAAAGGGCAGGCAGATCAC | ACCATATGCTTCAACATGTTCAA | 249 | Touchdown | g | 15 |
| **ARHGAP12 p.664** | TTGCAAGCTGTTCGTGAAAA | TCCCATCAACATCCAAACCATAT | 151 | Touchdown | c | 14-16 |

**Supplementary Table S2.** Clinical data for cats with primary hyperaldosteronism. Aldosterone reference intervals vary between laboratories 300 pmol/L is at or slightly higher than, the upper limit of the reference interval. Plasma renin activity is not widely available for cats so was not always measured. Systolic blood pressure (SBP) >160 mmHg is considered hypertensive. Normal electrolyte reference ranges depend on the sample type and analyzer used but are in the range of 149-158 mEq/L for sodium and 3.5-5.5 mEq/L for potassium. DSH; domestic short hair, DMH; domestic medium hair, DLH; domestic longhair, FN; female neutered, MN male neutered, [Aldo]; concentration of aldosterone, PRA; plasma renin activity, NA; not available, Pre-tx SBP; pretreatment systolic blood pressure, HT; hypertensive, HypoK; hypokalemic, Class; tumor classification, A; adenoma, Ax; adrenalectomy, PM; post mortem examination, ACC; adrenocortical carcinoma, Micro; Microscopic, distant mets; distant metastasis.

| Case code | Weight (kg) | Age (years) | Breed | Sex | [Aldo] (pmol/l) | PRA (ng/ml/hr) | Pre-tx SBP (mmHg) | Potassium (mEq/L) | Sodium (mEq/L) | Side | Source | Class | Mitotic rate (/10 HPF) | Largest diameter (cm) | Local invasion | Distant mets |
|---|---|---|---|---|---|---|---|---|---|---|---|---|---|---|---|---|
| 1 | 3 | 12.8 | DSH | FN | 725 | 0.84 | 180 | 3.0 | 155 | Right | Ax | ACC | 13 | 1.6 | Micro | NA |
| 2 | 5.7 | 13.4 | DLH | MN | 940 | NA | 158 | 2.0 | 155 | Left | Ax | A | 1 | 2.3 | None | None at PM |
| 3 | 6.1 | 13.3 | DLH | MN | 1371 | NA | NA | 2.5 | 155 | Right | Ax | ACC | 0 | 1.5 | Micro | NA |
| 4 | 4 | 14.8 | DSH | MN | >5000 | NA | 250 | 2.7 | 159 | Left | PM | ACC | 4 | NA | Gross | Lung |
| 5 | 3 | 14.1 | DSH | FN | >4605 | NA | 195 | 2.3 | 154 | Right | PM | ACC | 3 | 10 | Gross | None at PM |
| 6 | 4.5 | 13.6 | DMH | MN | 729 | 0.34 | 220 | 2.3 | 160 | Right | Ax | ACC | 6 | 1.9 | Micro | None on CT |
| 7 | 4.8 | 14.6 | DMH | FN | 1357 | NA | >200 | 2.7 | 161 | Left | Ax | ACC | 7 | 2 | None | NA |

| Case code | Weight (kg) | Age (years) | Breed | Sex | [Aldo] (pmol/l) | PRA (ng/ml/hr) | Pre-tx SBP (mmHg) | Potassium (mEq/L) | Sodium (mEq/L) | Side | Source | Class | Mitotic rate (/10 HPF) | Largest diameter (cm) | Local invasion | Distant mets |
|---|---|---|---|---|---|---|---|---|---|---|---|---|---|---|---|---|
| 8 | 8.5 | 14 | Siamese cross | MN | 2541 | NA | 165-300 | 2.3 | 155 | Left | Ax | A | 0 | NA | None | NA |
| 9 | 7.3 | 13 | DSH | FN | >4605 | NA | 250 | 3.2 | 158 | Right | PM | ACC | 1 | 4 | Micro | None at PM |
| 10 | 4.3 | 11.6 | DSH | MN | NA | NA | 220 | 2.5 | 152 | Right | Ax | A | 0 | 0.6 | None | None on CT |
| 11* | 5.7 | 9.8 | DSH | MN | 328 | NA | NA | 3.0 | 168 | Right | Ax | Adenoma | <1 | 2.3 | None | NA |
| 12 | 2.3 | 18 | DSH | FN | 790 | NA | NA | 2.2 | 150 | Left | PM | ACC | 1 | NA | Micro | None at PM |
| 13 | 3.8 | 7.2 | DLH | FN | 4416 | NA | 200-220 | 2.5 | 154 | Right | Ax | Adenoma | 1 | 1.7 | None | NA |

* Case 11: hypokalemia and hypertension resolved following adrenalectomy despite stopping all medications.

**Supplementary Table S3.** 67 somatic non-synonymous SNPs identified by whole genome sequencing (WGS), variants in bold were confirmed with bidirectional targeted sequencing. Location; Chromosome: locus, Amino acid; amino acid substitution, Count; Mean normalized count of RNA sequencing of all samples extracted from DeSeq2, Ref/Alt; reference/alternate allele count

| Case code | Location | Transcript | Gene | Amino Acid | SIFT | Count | Tumor reads (Ref/Alt) | Normal reads (Ref/Alt) |
|---|---|---|---|---|---|---|---|---|
| 1 | B4:35508185 | ENSFCAT00000037286.3 | **CACNA1C** | **G402D** | 0 | 3363 | 18/8 | 31/0 |
| 1 | D1:89166764 | ENSFCAT00000023724.3 | CSTF3 | R464Q | 0 | 350 | 17/12 | 30/0 |
| 1 | A2:13340908 | ENSFCAT00000007524.6 | IL12RB1 | V273F | 0 | 4 | 10/9 | 37/0 |
| 1 | D1:117264813 | ENSFCAT00000007588.5 | LRRC56 | K336T | 0 | 294 | 26/4 | 30/0 |
| 1 | E1:44324025 | ENSFCAT00000003051.6 | SLC4A1 | I540M | 0.01 | 1 | 14/4 | 26/0 |
| 1 | B2:92762811 | ENSFCAT00000030778.4 | MCHR2 | N274K | 0.02 | 0 | 29/4 | 38/0 |
| 1 | A2:9044680 | ENSFCAT00000000467.5 | TNPO2 | F882V | 0.03 | 1116 | 18/5 | 28/1 |
| 1 | C1:101348444 | ENSFCAT00000003469.6 | WDR3 | T48M | 0.03 | 987 | 12/11 | 22/0 |
| 2 | C1:142270545 | ENSFCAT00000048323.3 | KIF5C | F173C | 0 | 96 | 6/4 | 6/0 |
| 2 | C1:113563583 | ENSFCAT00000056623.2 | LIMS2 | F176V | 0 | 182 | 6/4 | 6/0 |
| 2 | D2:3817670 | ENSFCAT00000057872.2 | **PCDH15** | **D795Y** | 0 | 24 | 38/2 | 30/0 |
| 2 | D1:114419600 | ENSFCAT00000023485.4 | SHANK2 | E1416D | 0.01 | 223 | 6/4 | 6/0 |
| 2 | C1:120732877 | ENSFCAT00000029654.4 | PTPN4 | D636Y | 0.02 | 328 | 6/4 | 6/0 |
| 2 | C2:152303101 | ENSFCAT00000003470.6 | **CTNNB1** | **D32N** | 0.04 | 14951 | 6/4 | 6/0 |
| 3 | B4:134150255 | ENSFCAT00000002811.6 | BAIAP2L2 | N401T | 0 | 15 | 23/3 | 18/0 |
| 3 | D3:23713122 | ENSFCAT00000012688.5 | CD3H22orf31 | E221Q | 0 | 0 | 12/13 | 37/0 |
| 3 | B3:148801862 | ENSFCAT00000085815.1 | CEP170B | K1254N | 0 | 3669 | 17/3 | 39/0 |
| 3 | B2:32962667 | ENSFCAT00000004523.4 | **CYP21A2** | **L107R** | 0 | 131831 | 19/11 | 24/0 |
| 3 | C2:148986053 | ENSFCAT00000028845.4 | GADL1 | V269L | 0 | 0 | 19/12 | 28/0 |

| Case code | Location | Transcript | Gene | Amino Acid | SIFT | Count | Tumor reads (Ref/Alt) | Normal reads (Ref/Alt) |
|---|---|---|---|---|---|---|---|---|
| 3 | C1:204456007 | ENSFCAT00000024239.4 | GMPPA | L20V | 0 | 710 | 34/6 | 33/0 |
| 3 | C1:16086284 | ENSFCAT00000002965.6 | HSPG2 | G3031R | 0 | 9651 | 27/16 | 27/0 |
| 3 | E2:7856892 | ENSFCAT00000041910.2 | MYH14 | K1941T | 0 | 533 | 11/3 | 20/0 |
| 3 | D1:107792216 | ENSFCAT00000013687.6 | MYRF | C887W | 0 | 47 | 37/5 | 18/0 |
| 3 | E1:2490056 | ENSFCAT00000009621.5 | SOX15 | F56V | 0 | 1 | 20/4 | 33/0 |
| 3 | B2:4509987 | ENSFCAT00000072772.1 | CARMIL1 | K995Q | 0.01 | 1260 | 11/3 | 11/0 |
| 3 | E2:7856891 | ENSFCAT00000041910.2 | MYH14 | K1941Q | 0.01 | 533 | 11/3 | 21/0 |
| 3 | D4:63105983 | ENSFCAT00000008832.5 | ANKS6 | F96C | 0.02 | 206 | 12/4 | 10/0 |
| 3 | D4:19408723 | ENSFCAT00000027019.4 | **GNAQ** | **R183Q** | 0.03 | 499 | 16/12 | 33/0 |
| 3 | A2:5999468 | ENSFCAT00000065336.2 | MAP2K7 | T123P | 0.03 | 680 | 33/8 | 37/0 |
| 3 | D1:111580392 | ENSFCAT00000007392.6 | CCDC87 | F307V | 0.04 | 39 | 37/3 | 31/0 |
| 3 | F1:69342724 | ENSFCAT00000024156.4 | NES | L245V | 0.05 | 1965 | 8/4 | 13/0 |
| 4 | C2:133938732 | ENSFCAT00000001170.5 | ASTE1 | S617F | 0 | 160 | 26/16 | 24/0 |
| 4 | A1:216804816 | ENSFCAT00000000359.6 | CDH6 | K148M | 0 | 9 | 41/6 | 38/0 |
| 4 | A2:101760877 | ENSFCAT00000024460.4 | DLX6 | R181H | 0 | 0 | 29/19 | 24/0 |
| 4 | B3:54946054 | ENSFCAT00000010320.6 | GLDN | F544V | 0 | 407 | 249/8 | 32/0 |
| 4 | E3:9887814 | ENSFCAT00000060987.2 | HIP1 | E436D | 0 | 340 | 35/6 | 24/0 |
| 4 | E1:40189060 | ENSFCAT00000056807.2 | LASP1 | F30V | 0 | 4824 | 39/5 | 22/1 |
| 4 | B4:144057159 | ENSFCAT00000011916.6 | PLXNB2 | K419T | 0 | 4758 | 13/5 | 10/0 |
| 4 | C1:222067065 | ENSFCAT00000010453.6 | SNED1 | F62V | 0 | 695 | 16/8 | 17/1 |
| 4 | D4:82506838 | ENSFCAT00000023992.3 | STOM | Q148H | 0 | 3376 | 24/4 | 16/0 |
| 4 | D1:110283121 | ENSFCAT00000082838.1 | VPS51 | F218V | 0 | 1581 | 36/5 | 21/0 |
| 4 | B3:76258703 | ENSFCAT00000029851.4 | ZFHX2 | F870V | 0 | 380 | 566/20 | 20/0 |
| 4 | A3:36213561 | ENSFCAT00000023246.4 | ANKEF1 | R304W | 0.01 | 4 | 32/19 | 44/0 |

| Case code | Location | Transcript | Gene | Amino Acid | SIFT | Count | Tumor reads (Ref/Alt) | Normal reads (Ref/Alt) |
|---|---|---|---|---|---|---|---|---|
| 4 | D2:44875580 | ENSFCAT00000001652.4 | LRIT2 | Y521S | 0.03 | 0 | 37/4 | 21/0 |
| 4 | A1:72586838 | ENSFCAT00000006764.6 | ITGBL1 | D100A | 0.04 | 35 | 28/6 | 17/0 |
| 4 | D3:22598846 | ENSFCAT00000011935.5 | SF3A1 | M646K | 0.04 | 1455 | 580/40 | 12/0 |
| 4 | B3:76254453 | ENSFCAT00000029851.4 | ZFHX2 | T1047P | 0.05 | 380 | 476/21 | 24/0 |
| 5 | D4:94133256 | ENSFCAT00000050781.2 | ADAMTSL2 | Y136D | 0 | 239 | 52/4 | 13/0 |
| 5 | B4:29113884 | ENSFCAT00000038716.3 | **ARHGAP12** | **R664G** | 0 | 482 | 19/11 | 30/0 |
| 5 | X:126058991 | ENSFCAT00000054707.2 | CD99L2 | A149V | 0 | 655 | 13/18 | 34/0 |
| 5 | E3:4998042 | ENSFCAT00000011365.6 | DAGLB | S348G | 0 | 822 | 21/7 | 22/0 |
| 5 | X:127261840 | ENSFCAT00000018996.5 | GABRA3 | R481S | 0 | 0 | 23/15 | 33/0 |
| 5 | D2:89712785 | ENSFCAT00000011582.6 | KNDC1 | E1512K | 0 | 259 | 9/7 | 20/0 |
| 5 | D4:91141919 | ENSFCAT00000005119.6 | LAMC3 | N815T | 0 | 1127 | 63/5 | 29/0 |
| 5 | A1:213026293 | ENSFCAT00000027251.4 | LMBRD2 | S442R | 0 | 552 | 8/8 | 23/0 |
| 5 | A2:99838166 | ENSFCAT00000035004.3 | PPP1R9A | D189H | 0 | 715 | 23/8 | 30/0 |
| 5 | B4:85773017 | ENSFCAT00000000192.6 | R3HDM2 | L154P | 0 | 2016 | 37/3 | 27/0 |
| 5 | B4:78955711 | ENSFCAT00000041191.3 | SMARCD1 | I392S | 0 | 1095 | 33/3 | 19/0 |
| 5 | D1:16400271 | ENSFCAT00000001147.6 | BCL9L | L381V | 0.01 | 1011 | 16/3 | 18/1 |
| 5 | B2:121884680 | ENSFCAT00000069129.1 | CCN2 | Y250D | 0.01 | 385 | 16/3 | 31/1 |
| 5 | D1:109581669 | ENSFCAT00000076167.1 | PLCB3 | K469T | 0.01 | 570 | 9/5 | 18/1 |
| 5 | B2:11180517 | ENSFCAT00000040203.3 | RNF144B | S318P | 0.01 | 331 | 30/3 | 31/0 |
| 5 | C1:220683627 | ENSFCAT00000046646.3 | HDAC4 | F651V | 0.02 | 1679 | 27/7 | 30/0 |
| 5 | E2:9094174 | ENSFCAT00000025287.4 | NTN5 | H179P | 0.02 | 7 | 15/6 | 22/0 |
| 5 | F1:69778520 | ENSFCAT00000052992.2 | MEX3A | F370V | 0.03 | 24 | 16/4 | 19/0 |
| 5 | B4:144198620 | ENSFCAT00000067941.1 | SBF1 | I1361L | 0.03 | 2412 | 22/4 | 24/0 |
| 5 | E1:2542355 | ENSFCAT00000065820.2 | TP53 | V238M | 0.03 | 389 | 2/8 | 23/0 |

**Supplementary Table S4.** Exonic somatic non-synonymous single nucleotide polymorphisms (SNP), copy number variants (CNV) and insertion/deletions (InDel) detected by whole genome sequencing of adenoma (n=1) and adenocarcinomas (ACCs, n=4). For ACCs results are reported as median [range].

| | **Non-synonymous SNP (any SIFT)** | **Non-synonymous SNP (SIFT≤0.05)** | **CNV** | **InDel** |
|---|---|---|---|---|
| Adenoma (n=1) | 24 | 6 | 43 | 1 |
| ACC (n=4) | 40 [25-55] | 17 [8-20] | 369 [123-610] | 5 [4-6] |

**Supplementary Table S5.** Copy number variants (CNVs) present in more than one sample, detected by whole genome sequencing. Genes which are named within affected regions are annotated, and CNVs in each sample are reported. *Dup; identical duplication event, Dup; duplication in affected samples, Dup/Del; duplication and deletion event, Chrom; chromosome, POS1; start of CNV, POS2; end of CNV.

| Type | Chrom | POS1 | POS2 | Gene Names | Case1 | Case2 | Case3 | Case4 | Case5 |
|---|---|---|---|---|---|---|---|---|---|
| *Dup | B1 | 45612000 | 45647999 | ADAM32 | - | - | 3 | - | 3 |
| *Dup | B2 | 4364000 | 4399999 | HIST1H2BA,HIST1H2AA | 3 | - | 3 | - | - |
| *Dup | C2 | 133464000 | 133499999 | CPNE4 | - | - | 3 | - | 3 |
| *Dup | C2 | 159064000 | 159099999 | SUSD5 | - | - | 3 | - | 3 |
| Dup | F1 | 19156000 | 19187999 | TNN | 26 | - | 3 | - | - |
| Dup | C2 | 67224000 | 67255999 | STXBP5L | 5 | - | 3 | - | - |
| Dup | A3 | 45004000 | 45039999 | SLC24A3 | 9 | - | 3 | - | - |
| Dup | A3 | 54952000 | 54987999 | SULT1C3 | 4 | - | - | - | 3 |
| Dup | X | 105452000 | 105507999 | - | 31 | - | 4 | - | - |
| Dup | D2 | 90160000 | 90186660 | SYCE1 | - | - | 5 | - | 3 |
| Dup | E3 | 43536000 | 43587999 | CACNA1H | - | - | 5 | - | 4 |
| Dup | C1 | 156156000 | 156191999 | GRB14 | 55 | - | 7 | - | - |
| Dup | D2 | 80628000 | 80651999 | - | - | 3 | 12 | - | - |
| Dup/Del | F2 | 85732000 | 85752456 | - | 3 | - | - | - | 1 |
| Dup/Del | A1 | 162656000 | 162695999 | - | - | 10 | - | 1 | - |
| Dup/Del | B1 | 99140000 | 99175999 | - | - | 10 | - | 1 | - |
| Dup/Del | B4 | 100440000 | 100479999 | - | - | 18 | - | - | 0 |
| Dup/Del | B4 | 134872000 | 135187999 | CBX7, APOBEC3Z3, PDGFB, RPL3, SYNGR1, TAB1 | 1 | - | - | - | 3 |
| Dup/Del | E1 | 26916000 | 26951999 | - | - | 9 | - | - | 1 |
| Dup/Del | MT | 0 | 17009 | ND2, CXx1, COX2, ATP6, COX3, ND3, ND4, ND5, ND6, CYTB | - | 14 | - | 1 | 0 |

**Supplementary Figure S1.** Principle component analysis (A) showing gene expression from RNA sequencing of aldosterone secreting adrenal tumours from cats classified as adenomas (n=2, green) and carcinomas (n=3, blue), showing minimal separation by tumour classification. Point shape depicts analysis batch (circle; 1, triangle; 2). Volcano plot (B) showing 595 genes that were significantly differentially expressed(adjusted p <0.05). Grey points p>0.05 and $Log_2$ FC<2, green points p>0.05 and $Log_2$FC>2, blue points p<0.05 and $Log_2$ FC<2, red points p<0.05 and $Log_2$ FC>2.  NS; not significant, FC; Fold change.

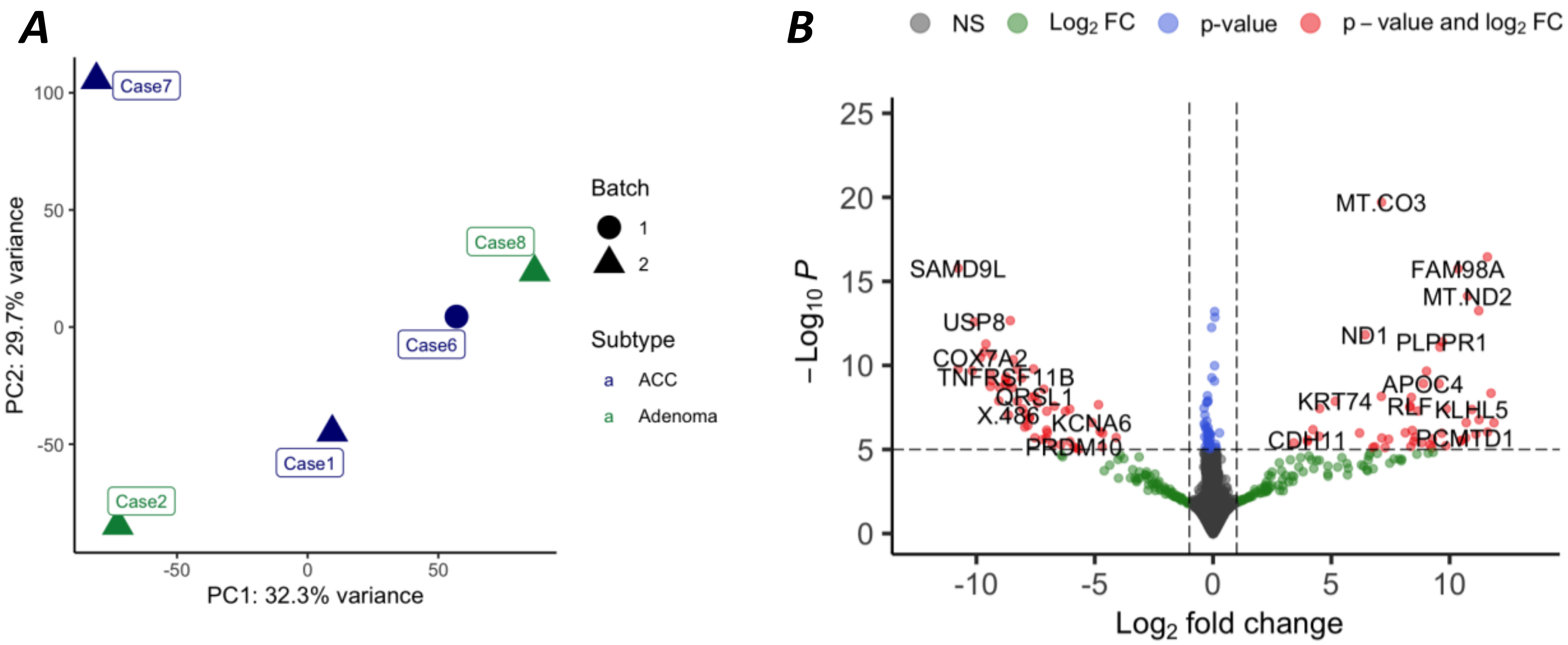

Supplement: Supplementary file 1 [file hyp-81-2489-s002.pdf]
